# Identification of ALDH2 as a novel target for the treatment of acute kidney injury in kidney transplantation based on WGCNA and machine learning algorithms and exploration of its potential mechanism of action using animal experiments

**DOI:** 10.3389/fimmu.2025.1536800

**Published:** 2025-03-04

**Authors:** Jinpu Peng, Shili Wang, Xingyu Pan, Moudong Wu, Xiong Zhan, Dan Wang, Guohua Zhu, Wei Wang, Hongyu Tang, Nini An, Jun Pei

**Affiliations:** Department of Pediatric Surgrey, Guizhou Provincial People’s Hospital, Guiyang, China

**Keywords:** kidney transplantation, acute kidney injury, renal ischemia-reperfusion injury, ALDH2, machine learning

## Abstract

**Background:**

Acute kidney injury (AKI) after kidney transplantation is one of the main causes of graft loss and poor patient prognosis, and it is important to explore new targets for treating AKI in kidney transplantation.

**Methods:**

Based on the kidney transplantation AKI-related dataset GSE30718, the most relevant modular genes for AKI among them were firstly screened using WGCNA and intersected with the DEGs, and the intersected genes were used as candidate genes for kidney transplantation AKI. Second, machine learning algorithms were utilized to identify the key genes among them, and the HPA database was used to explore the expression landscape. Next, we constructed a rat renal IRI model and explored the role of key genes in renal IRI. Finally, we combined ssGSEA enrichment analysis with animal experiments to further validate the potential mechanism of action of key genes.

**Results:**

In total, we identified 98 of the most relevant modular genes for AKI and 417 DEGs, which intersected to yield a total of 24 AKI candidate genes. Next, we intersected the key genes identified by three types of machine learning, namely, Random Forest, LASSO regression analysis and SVM, and obtained a total of 1 intersected gene as ALDH2, which we used as a key gene in kidney transplantation AKI. Using the HPA database, we found that ALDH2 has a high expression level in renal tissues and is mainly located in renal tubular epithelial cells. Next, we found in a rat renal IRI model that increasing the expression of ALDH2 alleviated the impairment of renal function and decreased the expression of NGAL, a marker of tubular injury, and BAX, an apoptotic protein, as well as reducing the expression of the inflammatory factors IL1β and IL6. Finally, using ssGSEA enrichment analysis and animal experiments, we further found that ALDH2 was able to inhibit the activation of the MAPK signaling pathway.

**Conclusion:**

ALDH2 may serve as a novel target for the treatment of kidney transplantation AKI, and increasing the expression level of ALDH2 has a protective effect on renal IRI, and this protective effect may be achieved by inhibiting the MAPK signaling pathway.

## 1 Introduction

Kidney transplantation is the best therapeutic option for patients with end-stage renal disease (ESRD), with better quality of life and lower mortality compared to dialysis patients (1). As immunosuppressive therapy techniques continue to evolve, graft survival rates have increased dramatically. Nonetheless, there are still some patients who develop short- or long-term complications after kidney transplantation, and in severe cases, the patients’ lives are even jeopardized (2). Acute kidney injury (AKI) is considered to be one of the major causes of acute and critical illness in kidney transplantation patients after surgery. This is because the development and progression of AKI after kidney transplantation is not only strongly associated with higher mortality, but also contributes to the development of later chronic diseases, such as chronic kidney disease (CKD) and chronic cardiovascular disease (3, 4). Not only that, but the occurrence of AKI will also greatly increase the risk of delayed recovery of graft function (DGF) and graft loss.

So far, ischemia-reperfusion injury (IRI) of varying degrees during kidney harvesting and transplantation has been recognized as one of the main causes of AKI after kidney transplantation (5). Many molecular and cellular alterations can be observed during IRI, such as the production of reactive oxygen species (ROS), cytokines, chemokines, activation of the innate immune system, activation of the inflammatory response, leukocyte recruitment, and alterations in a large number of biological regulatory pathways (6). This ultimately results in damage to endothelial cells and renal tubular epithelial cells, leading to the development of AKI after kidney transplantation. Meanwhile, it has also been found that AKI due to IRI during transplantation is likewise one of the key triggers of acute rejection after kidney transplantation (7). Therefore, taking treatment for IRI during kidney transplantation is an important measure to protect renal function, and the identification and exploration of new targets are of great significance for treatment.

With the wide availability of high-throughput sequencing technology, new approaches are provided for the identification of AKI-related biomarkers after kidney transplantation. The wide application of machine learning algorithms plays an important role in solving complex problems in the biomedical field. Machine learning’s ability to analyze large datasets and discover valuable relationships makes it an effective tool for elucidating patterns and providing explanations (8). Integrating bioinformatics analysis and machine learning with each other is important to improve the accuracy, reliability and predictability of disease diagnosis (8).

In the present study, we used the kidney transplantation AKI-related dataset GSE30718 as the basis, and screened the candidate genes among them that are closely related to AKI using the weighted gene co-expression network (WGCNA). Three machine learning algorithms, Random Forest, LASSO and SVM, were used to identify the key genes among them. We also explored the potential biological functions of key genes during kidney transplantation AKI. Finally, we constructed a rat kidney IRI model and explored the role of key genes and potential regulatory mechanisms using animal experiments. Provides new insights for future mitigation of kidney transplantation AKI.

## 2 Materials and methods

### 2.1 Data acquisition and processing

We obtained the kidney transplantation acute kidney injury-related dataset GSE30718 (GPL570, Affymetrix Human Genome U133 Plus 2.0 Array) via the GEO database (Gene Expression Omnibus, http://www.ncbi.nlm.nih.gov/geo). These included 8 cases of normal kidneys (Normal) and 28 cases of acute kidney injury kidneys (AKI). The Normal group was derived from normal renal tissue samples from the cortical region of eight patients with renal tumors that were not affected by pathology (9, 10). Next, we normalize the expression matrix of the dataset GSE30718 by “Sangerbox” (www.sangerbox.com). Sangerbox is a web-based tool platform that provides interactive and customizable analysis tools, including a variety of correlation analyses, pathway enrichment analyses, weighted correlation network analyses, and other common tools and features (11).

### 2.2 Weighted gene co-expression network analysis (WGCNA) and key module gene identification

In the present study, we used “WGCNA analysis” in “OECloud (www.cloud.oebiotech.cn/) “ to identify the most relevant modular genes for kidney transplantation AKI in the dataset GSE30718. We entered the expression profiles of the dataset GSE30718 into “WGCNA” of “OECloud”, and also entered the grouping information of the samples as required. Subsequently, we set the standard deviation threshold to 0.5, the module merge threshold to 0.25, the minimum number of genes to 20, and the segmentation sensitivity to 2. After running “OECloud”, genes with similar expression patterns will be categorized into the same module under the optimal soft threshold conditions. Finally, we select the module with the highest absolute value of the correlation coefficient as the key module for subsequent analysis. WGCNA is a common tool often used to reveal genetic associations between different samples and to detect candidate therapeutic targets based on the inter-association of gene sets as well as the association between gene sets and phenotypes.

### 2.3 Identification of candidate genes for kidney transplantation AKI

In order to improve the accuracy of the key module genes, we firstly used the expression matrix of the dataset GSE30718 as the basis to obtain the differential genes between the AKI group and the Normal group by using the “Limma Rapid Difference Analysis Tool” in the Sangerbox platform. Log2Fold absolute value >1.0 and adj. P<0.05 were used as the criteria for identifying differential genes (DEGs). Next, we intersected key module genes with DEGs, and the intersected genes we defined as kidney transplantation AKI candidate genes and used them for the next step of analysis.

### 2.4 Identification of Hub genes using machine learning algorithms

In this study, we utilized three machine learning algorithms to characterize the Hub gene.

In the first one, we identify the key genes through the “Random Forest” algorithm in “OECloud”. We entered the expression profiles of the kidney transplantation AKI candidate genes into the “Random Forest” in “OECloud” and entered the grouping information of the samples as required. “OECloud” automatically runs the Random Forest algorithm and judges how well the genes classify the model according to the MeanDecreaseGini value. Where a larger MeanDecreaseGini value represents a better categorization and the final results are sorted according to the size of the MeanDecreaseGini value. We define the top 5 genes as “Random Forest-Key Genes”.

Second, we identified key genes by “LASSO regression analysis” in “Sangerbox”. We entered the expression profiles of the kidney transplantation AKI candidate genes into the “LASSO-regression analysis tool” in “Sangerbox”, and entered the grouping information of the samples. The “Sangerbox” platform automatically runs the R package glmnet, which integrates grouping information and gene expression profiling data for “LASSO-regression analysis”. Meanwhile, the “Sangerbox” platform has set up a 3-fold cross-validation in order to obtain the optimal model. We chose the best lambda value to construct the “LASSO-regression analysis” model, and we defined the genes with coefficient value not equal to 0 as “LASSO- Key Genes” in this model.

In the third one, we utilized the “SVM” algorithm in the “Wekemo Blolncloud Platform” (https://www.bioincloud.tech) to identify key genes. We entered the expression profiles of kidney transplantation AKI candidate genes into the “Wekemo Blolncloud Platform” and normalized the expression profiles. The standardized correction is divided into three main steps: 1. Within-sample correction, i.e., the abundance of all FEATURES (genes) in a sample is divided by the median abundance of that sample; 2. abundance matrix correction, i.e., log transformation of all abundance values; 3. within-feature correction, i.e., abundance of all samples corresponding to a feature minus the mean abundance of that feature divided by the standard deviation of abundance of that feature. Next, we ran the “SVM” algorithm in the Wekemo Blolncloud platform and ranked the genes according to their contribution to the group differences, and defined the top 5 genes as “SVM- Key Genes”.

The above three machine learning algorithms have been used in our previous studies (12, 13). Finally, we intersect the key genes identified by the three machine learning algorithms described above, and the intersecting genes we define as Hub genes.

### 2.5 Hub gene expression landscape

We used the Human Protein Atlas database (HPA: https://www.proteinatlas.org/) to explore the expression landscape of Hub genes in various tissues and organs. The “Single Cell” module of the HPA database was also utilized to study the extent of Hub gene expression at the cellular level. The results were all generated from the HPA database. The HPA Database is a publicly accessible and freely explorable database of all human proteins in cells, tissues, and organs mapped with the integration of various histological techniques, including antibody-based imaging, mass spectrometry-based proteomics, transcriptomics, and systems biology.

### 2.6 ssGSEA enrichment analysis

ssGSEA can analyze the pathways that are enriched for gene expression in each sample. ssGSEA requires pre-selection of a biologically significant set of genes compared to traditional GSEA enrichment analysis, and then genes with the same significance or function within the set of genes are computed together and grouped into a single enrichment score to analyze the degree of activation of a specific pathway (14). In this study, in order to understand the changes of all the KEGG-normalized pathways during kidney transplantation AKI, we used the ssGSEA enrichment algorithm in the Sangerbox online analysis tool to enrich 186 pathways in the “C2:KEGG gene-sets” to explore the changes of the relevant pathways during kidney transplantation AKI. At the same time, we calculated the correlation between 186 pathways and Hub genes by “sample correlation analysis” in “OECloud”, and the results were shown in a lollipop plot. p<0.05 was considered statistically significant.

### 2.7 Establishment of animal models

We selected a total of 20 adult male Sprague-Dawley (SD) rats, purchased from the SPF (Beijing) Biotechnology Co., Ltd. (SCXK[JING] 2024-0001, Beijing, China), weighing 250-280g. The animal model was taken as a renal ischemia reperfusion injury (IRI) model, as it has been noted that AKI during kidney transplantation is mainly due to IRI of the kidney during transplantation (5). All rats were randomly divided into four groups of 5 rats each, namely, Sham group, Sham+Alda-1 group, IRI group and IRI+Alda-1 group. The Sham+Alda-1 and IRI+Alda-1 groups were treated with rat Alda-1 (20 mg/Kg) by intraperitoneal injection once daily for 3 days. Alda-1 is a selective agonist of ALDH2 and can effectively increase the expression level of ALDH2 (15). Rats in the Sham and IRI groups were treated with equal doses of saline intraperitoneally. On day 4, after the rats were routinely anesthetized, skinned, disinfected and toweled, all rats had their right kidneys removed first. Subsequently, rats in the IRI group and IRI+Alda-1 group were bluntly isolated from the left renal hilum, and the left renal hilum was clamped using a noninvasive vascular clip for 45 min, and the color of the kidneys was observed to change from bright red to purplish-black, suggesting that renal ischemia was successful. After releasing the vascular clamp, the kidney color was observed to gradually return to bright red, indicating successful reperfusion. In contrast, rats in the Sham and Sham+AIda-1 groups had only blunt separation of the left renal hilum, but were not treated for ischemia. After 24 hours, four groups of rats were killed suddenly under anesthesia, and venous blood was taken to test the renal function of the rats in each group, and the left kidney was taken for subsequent experiments. Animal experiments were approved by the Animal Ethics Committee of Guizhou Provincial People’s Hospital, and all animal studies followed the ARRIVE guidelines.

### 2.8 Renal function tests

Under anesthesia, about 3 ml of blood was taken from the lower vena cava of rats, and the upper serum was centrifuged and placed in an automatic biochemical analyzer after 15 min of resting, and the levels of urea nitrogen and creatinine were detected in each group of rats, which were used to evaluate the renal function damage of rats in each group.

### 2.9 Western blot

Proteins in rat kidney tissue were extracted with RIPA lysate containing protease inhibitors, and protein concentration was determined by BCA kit (Beyotime, No.P0012). Equal amounts of protein samples were separated by SDS-PAGE and then transferred to PVDF membranes, which were closed and incubated with primary antibodies overnight at 4°C, all at a dilution concentration of 1:1000. On the second day, after washing the membrane with TBST, the secondary antibody was incubated at room temperature for 1 h. The secondary antibody was diluted at a concentration of 1:15,000. Finally, Western blot bands were analyzed semi-quantitatively using Image J software, and P < 0.05 was considered significantly different. Primary antibodies were purchased from the following sources: anti-ALDH2 (ABclonal, A1226), anti-NGAL (ABclonal, A2092), anti-BAX (ABclonal, A0207), anti-IL-6 (Abcam, ab290735), anti-IL1-β (Abcam, ab315084), anti-P38 (ABclonal, A5049), anti-p-P38 (ABclonal, AP0057), anti-ERK (ABclonal, A22447), anti-p-ERK (ABclonal, AP0485), anti-JNK (Proteintech, 28007-1-AP) and anti-p-JNK (ABclonal, AP0631).

### 2.10 Statistical analysis

Image J and Prism software (GraphPad Software, La Jolla,CA) were used for statistical analysis of experimental data. Differences between the two groups were analyzed using the unpaired Student’s t-test. One-way ANOVA was used for multiple group comparisons. **** represents P<0.0001, *** represents P<0.001, ** represents P<0.01, and * represents P<0.05.

## 3 Results

### 3.1 Identification of key modular genes for kidney transplantation AKI

**Figure 1:**
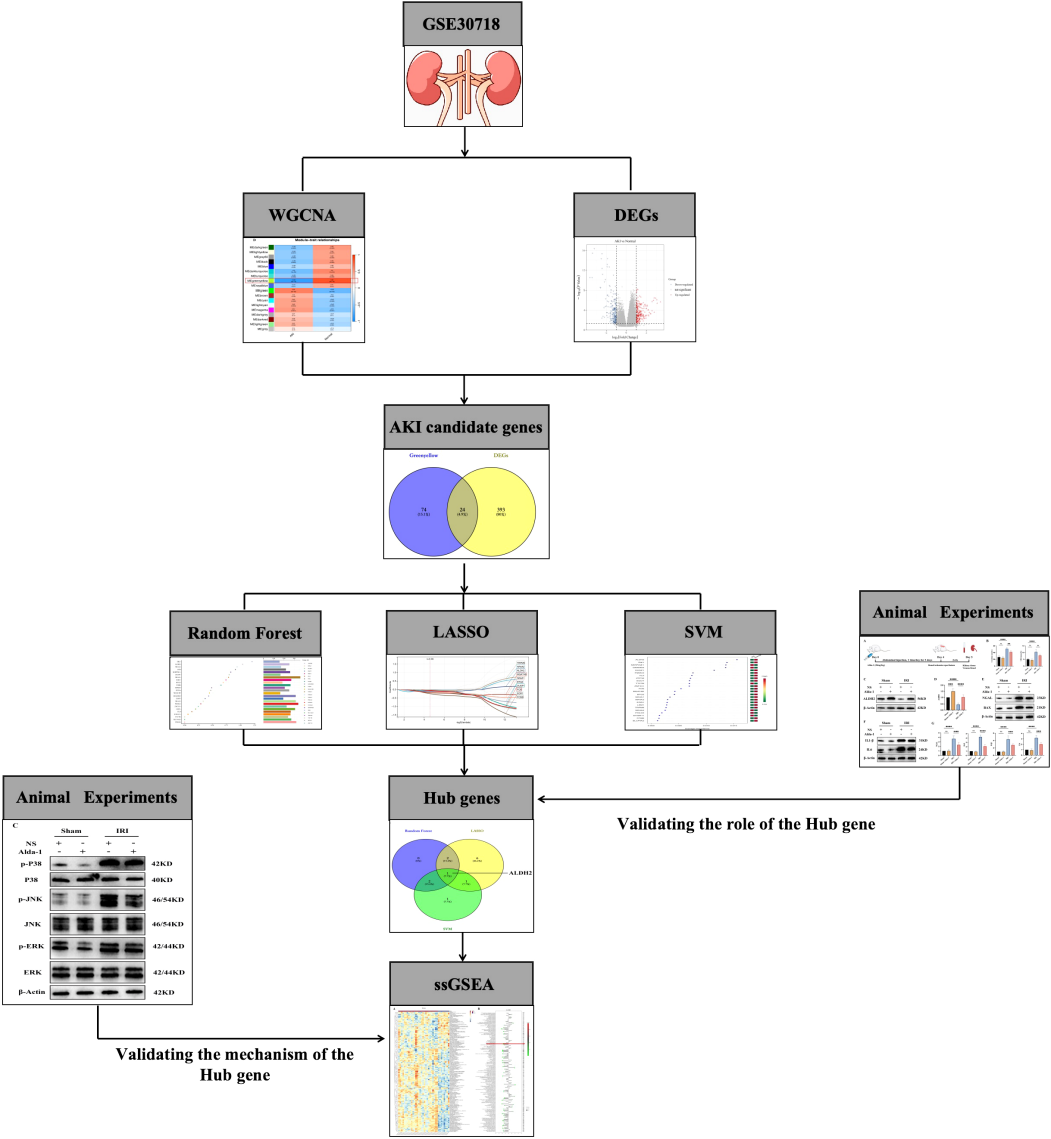
Flowchart of the current study.

**Figure 2:**
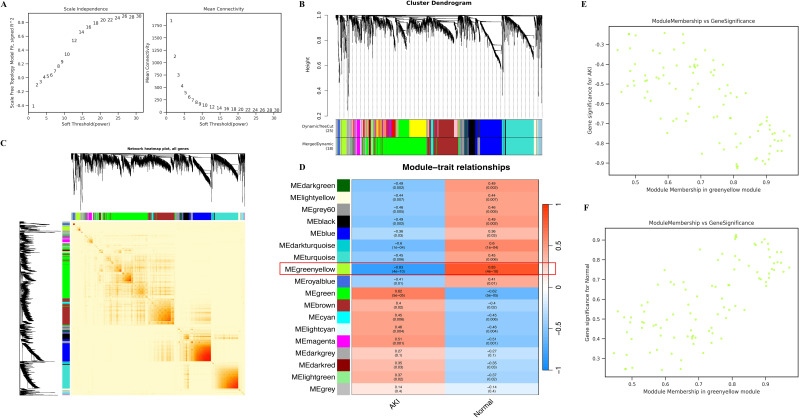
Identification of key modules using WGCNA. **(A)** represents the determination of the optimal β value using a scale-free topological model, with β = 20 chosen as a soft threshold based on average connectivity and scale independence; **(B)** represents the gene clustering dendrogram; **(C)** represents a heat map of gene clustering; **(D)** represents a heat map of the correlation between different modules and features; **(E)** represents the scatter plot of the correlation between AKI and greenyellow module, and the results show that AKI is negatively correlated with greenyellow module; **(F)** represents the scatter plot of the correlation between the Normal and greenyellow modules, and the results show that the Normal and greenyellow modules are positively correlated.

The flow chart of this study is shown in **Figure 1**. We further identified AKI-related modular genes in the dataset GSE30718 using the weighted gene co-expression network (WGCNA). A total of 18 modules were identified when a soft threshold of 20 was chosen based on scale independence and average connectivity (**Figures 2A, D**), where the Grey module was a collection of genes that could not be attributed to any of the modules and had no reference (**Figure 2D**). The genes are clustered as shown in **Figure 2B**, and the clustered heatmap of the genes is shown in **Figure 2C**. Next, we find that the greenyellow module has the highest correlation, with an absolute value of 0.83 for the correlation coefficient. Which was negatively correlated with AKI and positively correlated with Normal (**Figure 2D**), and scatter plots of correlation analysis of traits with modular genes similarly confirmed these findings (**Figures 2E, F**). This implies that most of the genes in the greenyellow module were highly expressed in the Normal group and lowly expressed in the AKI group, and that the reduced expression of these genes may be closely related to the development of AKI. Increasing the expression levels of these genes may be able to be a potential target for alleviating AKI in kidney transplantation. Therefore, we used the genes in the greenyellow module for the next step of the analysis, which contained 98 genes.

### 3.2 Identification of AKI candidate genes for kidney transplantation

**Figure 3:**
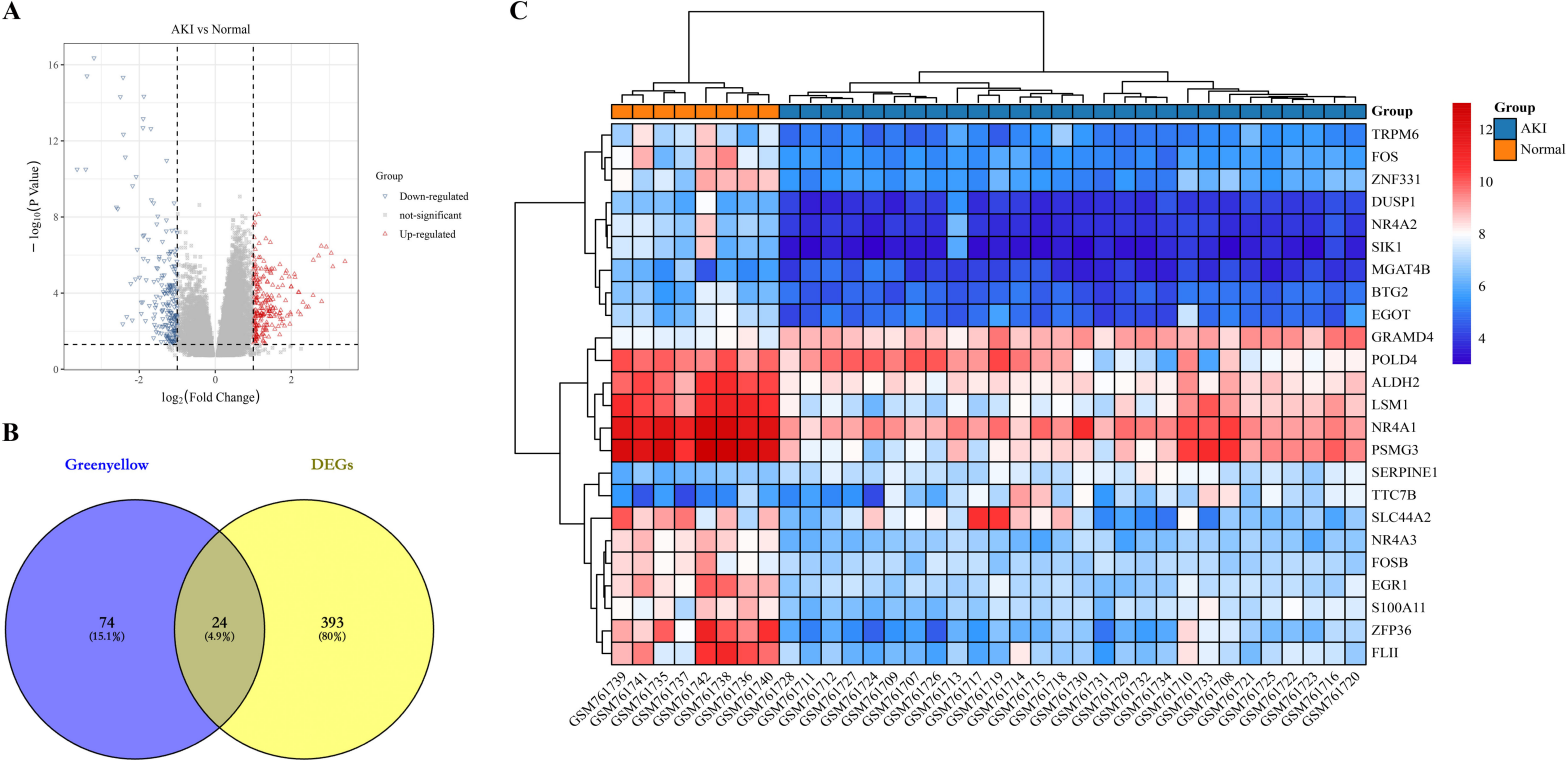
Identification of candidate genes for kidney transplantation AKI. **(A)** represents a volcano plot of gene expression differences in AKI compared to the Normal group in dataset GSE30718; **(B)** represents the Greenyellow module with DEGs intersecting Veen diagrams; **(C)** represents the expression heat map of the 24 intersecting genes.

First, we identified differential genes (DEGs) in the dataset GSE30718. We identified a total of 417 differentially expressed genes, of which 228 genes were up-regulated and 189 genes were down-regulated in AKI compared to Normal (**Figure 3A**). Next, we intersected the 417 DEGs with the 98 greenyellow module genes and identified a total of 24 intersecting genes, which we defined as kidney transplantation AKI candidate genes (**Figure 3B**). The gene expression heat map is shown in **Figure 3C**.

### 3.3 Identification of Hub genes

**Figure 4:**
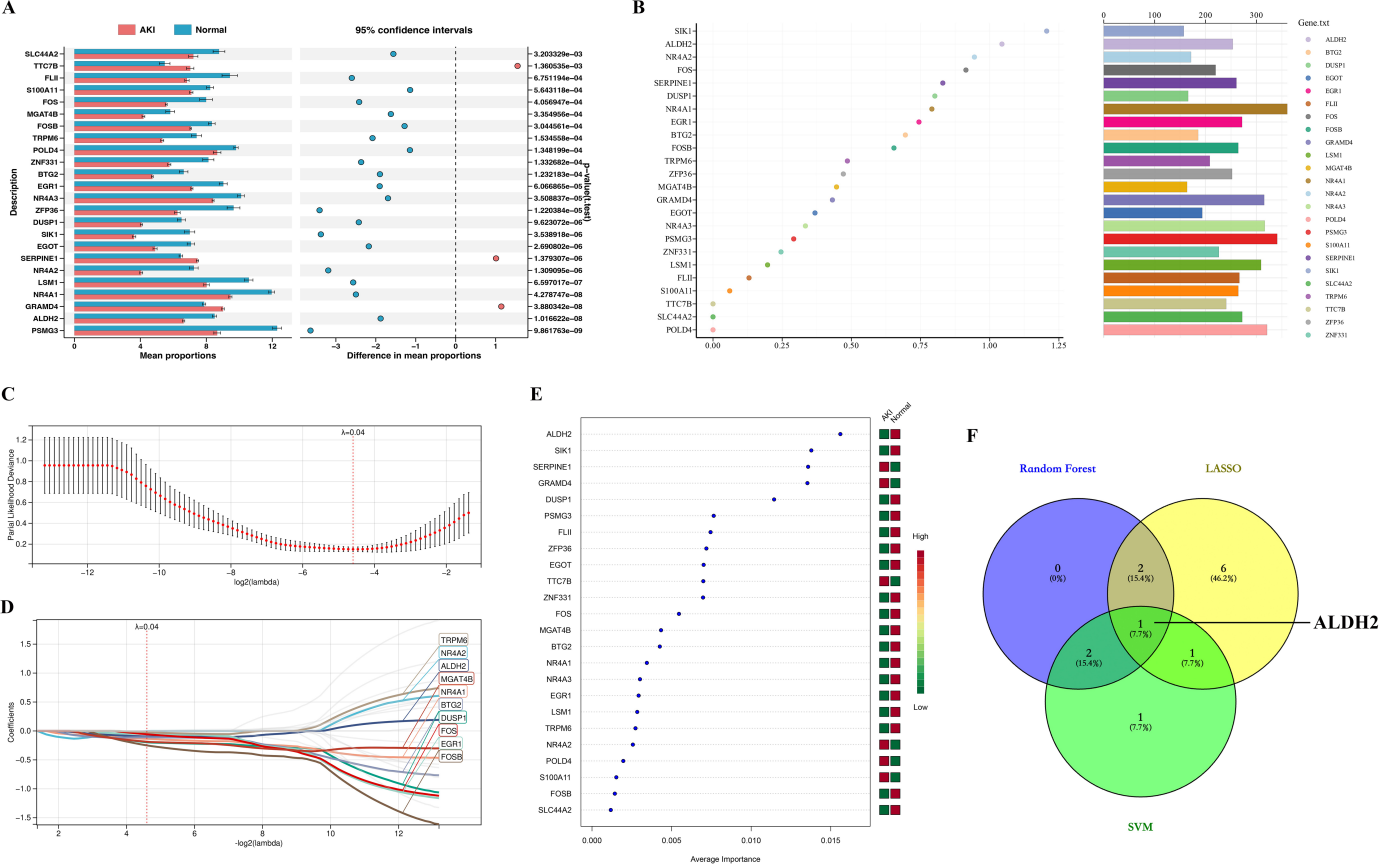
Identification of Hub genes. **(A)** represents a statistical plot of the expression levels of 24 genes between the AKI and Normal groups; **(B)** stands for Random Forest algorithm to identify key genes, where the x-axis represents the MeanDecreaseGini value and the bars on the right represent the relative abundance of genes; **(C)** represents the cross-validation error plot in the LASSO regression analysis. Where the x-axis represents the logarithmic value of lamabda and the y-axis represents the model deviation in cross-validation, the smaller the deviation, the better the model fit. Ultimately, we determined that 0.04 was the best lambda (ℷ) value. **(D)** represents the plot of the LASSO-regression analysis model constructed at ℷ = 0.04, where the colored curves are for genes with coefficient values not equal to 0 and the gray curves are for genes with coefficients equal to 0; **(E)** represents SVM algorithm to identify key genes. Where the squares on the right side represent two subgroups (AKI group and Normal), with green representing low gene expression and red representing high gene expression; **(F)** represents the intersection veen plot of key genes identified by the three machine learning algorithms.

In the present study, we first statistically analyzed the expression levels of the 24 genes mentioned above. We found that the expression of 21 of these genes was significantly lower in the AKI group, and only TTC7B, SERPINE1, and GRAMD4 were significantly higher in the AKI group than in the Normal group (**Figure 4A**). This is consistent with the expressive properties of the greenyellow module. Next, we utilized three machine learning algorithms to identify key genes.

In the Random Forest algorithm, we ranked the genes according to the size of the MeanDecreaseGini value, and we defined the top 5 genes as the Random Forest key genes: SIK1, ALDH2, NR4A2, FOS and SERPINE1 (**Figure 4B**).

In the “LASSO-regression analysis” algorithm, through triple-fold cross-validation, we found that the error was minimized for lambda (ℷ) value = 0.04 (**Figure 4C**). Therefore, we constructed a “LASSO-regression analysis” model based on “ℷ = 0.04” (**Figure 4D**). Under this model, we identified a total of 10 genes with coefficient values not equal to 0, which we used as LASSO-key genes, namely, TRPM6, NR4A2, ALDH2, MGAT4B, NR4A1, BTG2, DUSP1, FOS, EGR1, and FOSB (**Figure 4D**).

In the “SVM” algorithm, we ranked the genes according to their contribution to the group differences, and the top 5 genes were defined as SVM- key genes: ALDH2, SIK1, SERPINE1, GRAMD4, and DUSP1 (**Figure 4E**).

Finally, we intersected the key genes identified by the three machine learning algorithms described above, and a total of one intersecting gene, ALDH2, was identified, which we defined as a Hub gene (**Figure 4F**).

### 3.4 Hub gene expression landscape analysis

In the present study, we explored the expression landscape of the Hub gene ALDH2 at the tissue organ and cellular levels using the HPA database. First, in terms of protein expression level, ALDH2 was highly expressed in renal tissues, and its expression level was located in the third position among all tissues and organs (**Supplementary Figure 1A**). In terms of mRNA expression levels, ALDH2 was likewise in the third position in renal tissues, after liver tissues and adipose tissues (**Supplementary Figure 1B**). Second, in terms of single-cell expression levels, ALDH2 was highly expressed in renal tubular epithelial cells, and its expression was only lower than that in hepatocytes (**Supplementary Figure 1C**). Single-cell sequencing of renal tissues similarly revealed that ALDH2 was predominantly located in proximal tubular epithelial cells for expression (**Supplementary Figure 1D**). Finally, we found that ALDH2 was predominantly expressed in the renal tubules in normal renal tissues by immunohistochemical results from the HPA database, in keeping with the previous results (**Supplementary Figure 1E**). From this, we determined that ALDH2 is a highly expressed protein located in renal tubular epithelial cells.

### 3.5 ALDH2 alleviate renal ischemia-reperfusion injury

**Figure 5:**
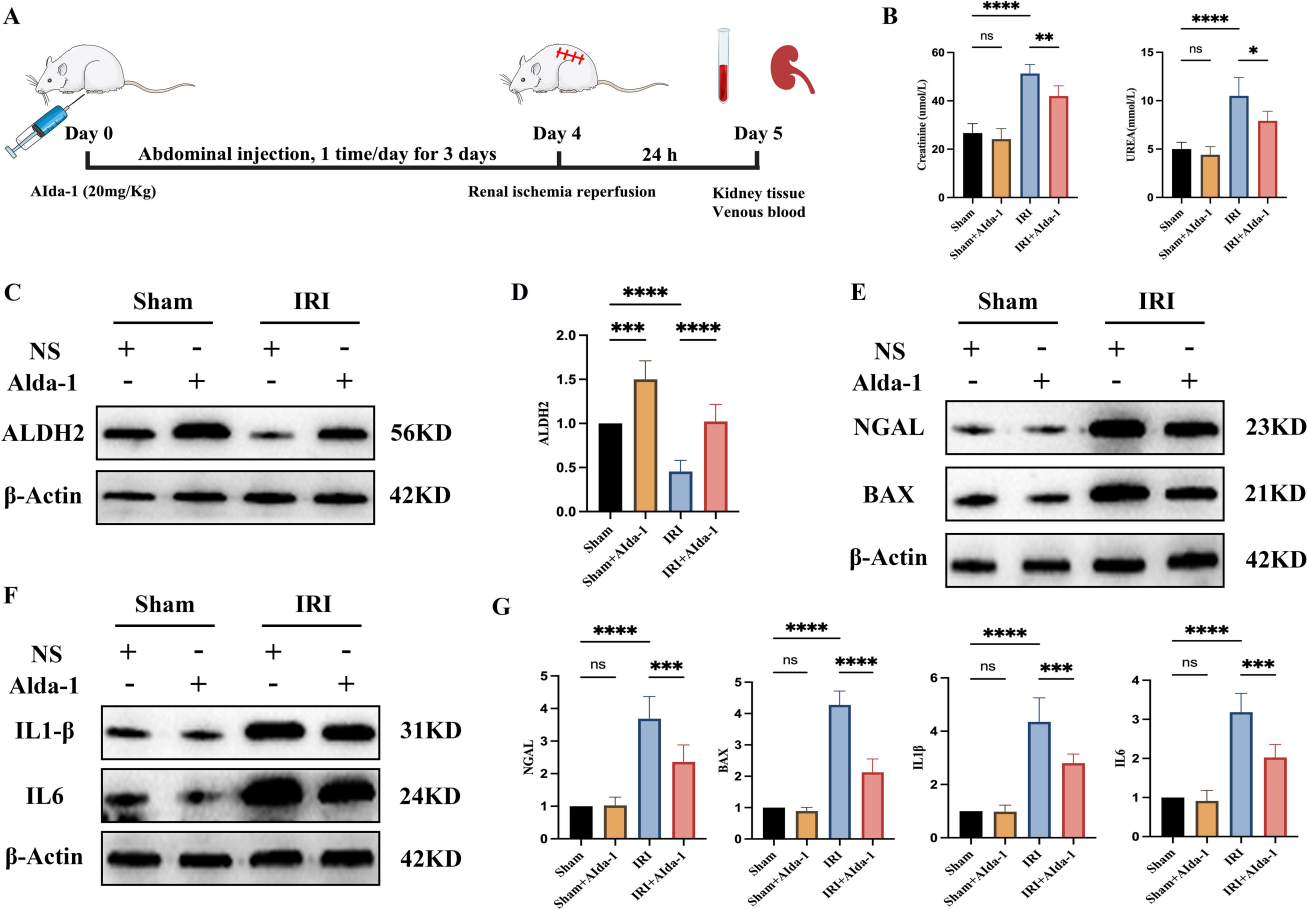
ALDH2 alleviates renal ischemia-reperfusion injury. **(A)** represents the flowchart of this experiment; **(B)** represents the expression level of renal function in each group of rats; **(C)** represents the Western blot strip plot of ALDH2 expression levels in rat kidney tissues; **(D)** represents the Western blot strip statistics of ALDH2 expression levels; **(E)** represents Western blot strip plots of NGAL and BAX expression levels in rat kidney tissues; **(F)** represents the Western blot strip plot of the expression levels of inflammatory factors (IL1β and IL6) in rat kidney tissues; **(G)** represents Western blot band statistics. **** represents P<0.0001, *** represents P<0.001, ** represents P<0.01, * represents P<0.05 and, ns represents P>0.05.

We intervened to increase the expression level of ALDH2 in renal tissues using Alda-1, a selective agonist of ALDH2, based on the rat renal IRI model in the present study. The flowchart for this experiment is shown in **Figure 5A**. We found that renal function was significantly impaired when the kidneys were subjected to IRI (**Figure 5B**), and ALDH2 expression levels were significantly reduced in renal tissues (**Figures 5C, D**), which is in keeping with the results of our previous analysis. When Alda-1 was administered, the expression level of ALDH2 in renal tissues was significantly increased (**Figures 5C, D**), demonstrating that Alda-1, as an agonist of ALDH2, can significantly increase the expression level of ALDH2 in renal tissues. Next, we found that the degree of impairment of renal function was significantly alleviated after increasing ALDH2 expression compared with the IRI group (**Figure 5B**). This implies that ALDH2 has a protective effect on renal function in rats. The expression levels of NGAL, a marker of renal tubular injury, and BAX, an apoptotic protein, were detected by Western blot, and it was found that the expression levels of NGAL and BAX were significantly increased in the IRI group compared with the Sham group. After being boosted ALDH2, the expression levels of NGAL and BAX were reduced compared with the IRI group, demonstrating that ALDH2 was able to alleviate the levels of injury and apoptosis in renal tubular epithelial cells (**Figures 5E, G**). This shows that ALDH2 has a protective effect on renal IRI.

### 3.6 ALDH2 alleviates inflammatory response during renal IRI

We examined the expression levels of inflammatory factors IL1β and IL6 in the renal tissues of rats in each group by Western blot in the present study. We found that the expression levels of inflammatory factors were significantly increased in the renal tissues of rats in the IRI group compared with the Sham group, demonstrating that ischemia-reperfusion injury induces an inflammatory response in the renal tissues (**Figures 5F, G**). When the expression of ALDH2 was increased, the expression levels of inflammatory factors IL1β and IL6 were significantly reduced in renal tissues compared with the IRI group, demonstrating that ALDH2 was able to reduce the secretion of inflammatory factors in the course of renal IRI (**Figures 5F, G**).

### 3.7 Potential mechanisms of action of ALDH2

**Figure 6:**
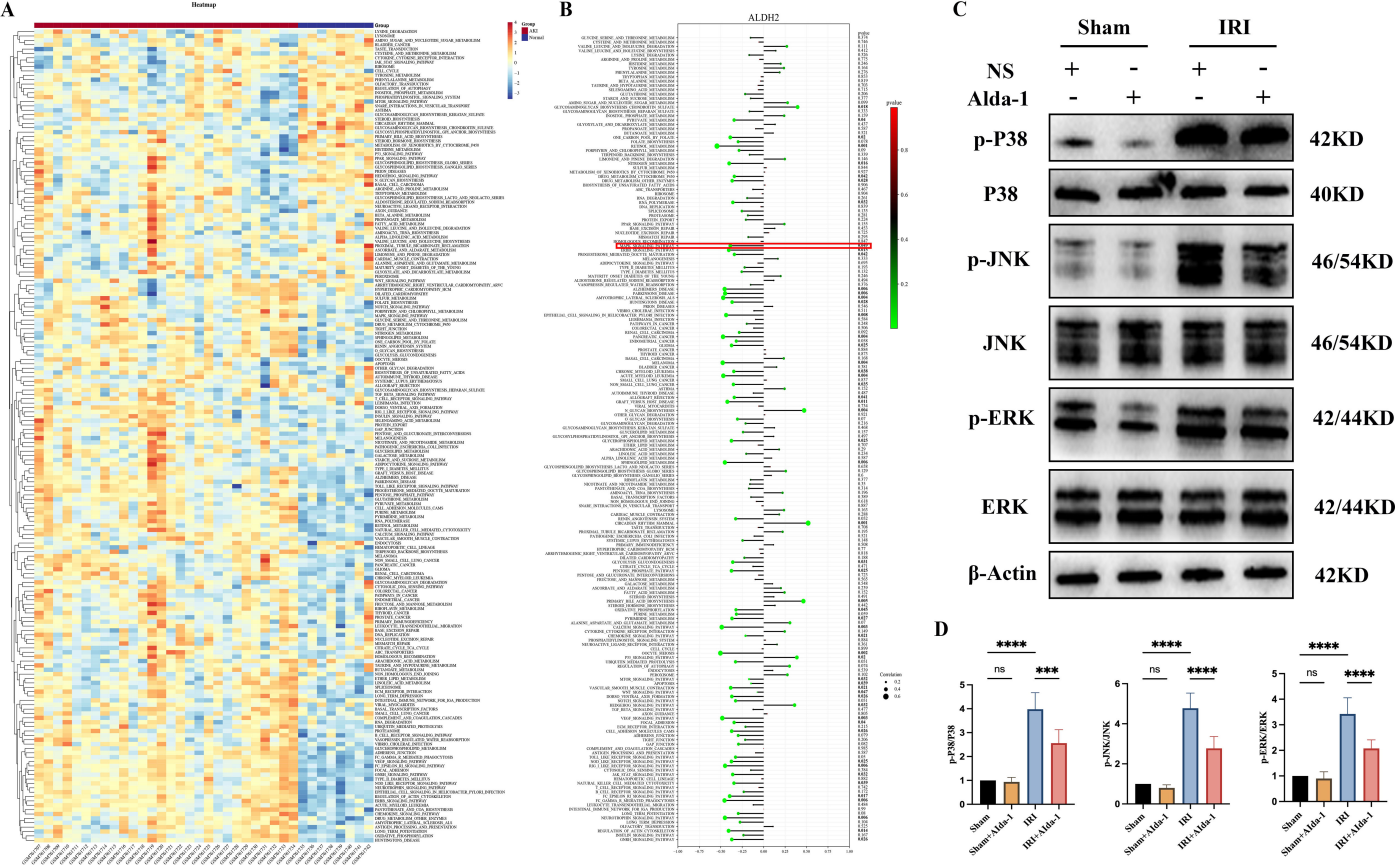
ssGSEA enrichment analysis and validation of the MAPK signaling pathway. **(A)** represents a heatmap of the expression levels of 186 KEGG signaling pathways between the AKI and Normal groups in dataset GSE30718; **(B)** represents a lollipop plot of the correlation analysis of the Hub gene ALDH2 with 186 KEGG signaling pathways; **(C)** represents the western blot bar graph of the expression level of MAPK signaling pathway in the kidney tissues of rats in each group; **(D)** represents the Western blot strip statistics of the MAPK signaling pathway. **** represents P<0.0001, *** represents P<0.001  and, ns represents P>0.05.

We found in the above results that increasing the expression of ALDH2 was effective in alleviating renal IRI, while also reducing the level of inflammation in renal tissues. To further explore the potential protective mechanism of ALDH2, we utilized ssGSEA to explore the biological functional differences during kidney transplantation AKI in the current study. We found that a large number of biological pathways were significantly altered between the AKI and Normal groups, which we demonstrated with heat maps (**Figure 6A**). To further analyze the potential regulatory mechanisms of ALDH2 during kidney transplantation for AKI, we explored the correlation between ALDH2 and the expression levels of 186 KEGG signaling pathways using correlation analysis. We found that ALDH2 was closely linked to the expression of a large number of signaling pathways during kidney transplantation for AKI (**Figure 6B**). Also, we found that ALDH2 was negatively correlated with the expression of most signaling pathways, implying that ALDH2 was able to inhibit biological pathways during kidney transplantation for AKI (**Figure 6B**). Among them, the MAPK signaling pathway has attracted our attention. In our previous study, we found that inhibition of the MAPK signaling pathway was effective in alleviating IRI in the kidney (16). In the present study, we found that ALDH2 expression was negatively correlated with the activation of the MAPK signaling pathway (**Figure 6B**). This implies that increasing the expression of ALDH2 inhibits the activation of the MAPK signaling pathway. Next, we examined the expression level of MAPK signaling pathway in the kidney tissues of rats in each group using Western blot. We found that the MAPK signaling pathway was significantly activated in the IRI group compared with the Sham group (**Figures 6C, D**). However, after the expression level of ALDH2 was increased, the expression level of the MAPK signaling pathway was significantly reduced (**Figures 6C, D**). This implies that ALDH2 is able to inhibit the expression level of the MAPK signaling pathway, which is consistent with the results of ssGSEA enrichment analysis. Therefore, we suggest that the protective effect of ALDH2 may be achieved by inhibiting the MAPK signaling pathway.

## 4 Discussion

Kidney transplantation is the treatment of choice for end-stage renal disease and likewise the most common type of organ transplantation available. During kidney transplantation, AKI caused by renal IRI is an unavoidable event that may trigger a series of pathophysiologic processes leading to delayed recovery of graft function (DGF), impaired graft function, and rejection (17). How to effectively mitigate kidney transplantation AKI is a great challenge that is being faced. With the current widespread use of high-throughput sequencing technology, it has provided tremendous help in exploring the molecular landscape and underlying mechanisms of disease (18). Combining sequencing data and comprehensive bioinformatics analysis techniques with each other provides sufficient theoretical basis for discovering new targets and mechanisms for disease treatment. We used WGCNA and machine learning algorithms to identify key genes in kidney transplantation AKI in the current study and investigated the expression landscape of key genes, correlation with immune cell infiltration, and biological pathways potentially regulated. Finally, we also used animal experiments to validate the roles and possible mechanisms of key genes. A new option for the future treatment of kidney transplantation AKI.

We first identified a total of 98 modular genes most closely associated with kidney transplantation AKI using WGCNA in the current study. Intersecting them with DEGs yielded a total of 24 genes, which we used as candidate genes associated with kidney transplantation AKI. When we analyzed the expression levels of 24 genes, we found that 21 of them had significantly lower expression in the AKI group, and only 3 genes had significantly higher expression in the AKI group than in the Normal group. This means that most of the candidate genes were negatively associated with the development of AKI in kidney transplantation, a result that is consistent with the analysis of WGCNA, where the module with the highest correlation coefficient was negatively associated with AKI. To further explore the key genes involved, we performed calculations using three machine learning algorithms (Random Forest, LASSO regression analysis, and SVM algorithm), and finally identified ALDH2 as the most critical gene for kidney transplantation AKI. Meanwhile, the expression level of ALDH2 in the AKI group was significantly lower than that in the Normal group, which may imply that increasing the expression level of ALDH2 may be a new target for alleviating AKI.

Aldehyde dehydrogenase-2 (ALDH2) is one of the members of the aldehyde dehydrogenase (ALDH) family, which is mainly responsible for the detoxification of biological and xenoaldehydes (19). ALDH is widely distributed and is present in bacteria, fungi and all eukaryotic organisms (19, 20). In the study of human genomics, 19 functional ALDH genes were found to be widely expressed in tissues, among which the role of ALDH2 attracted the attention of researchers (19). In humans, ALDH2 is a polypeptide containing 517 amino acids and is encoded by the cytosolic gene on chromosome 12q24 (21). Like most members of the ALDH family, ALDH2 is a tetrameric enzyme with ∼56 kDa that possesses not only dehydrogenase activity but also reductase and esterase activities (15, 19). ALDH2 is ubiquitously expressed in all tissues, with the most abundant expression levels in the liver, which was similarly confirmed when the ALDH2 expression landscape was studied through the HPA database, which is known for the key role that leads to the major involvement of ALDH2 in ethanol metabolism in the liver (15, 19). In addition to this, ALDH2 was similarly detected in large quantities in organs such as kidney (22), heart (23), brain (24), and lungs (25), reconfirming the prevalence of its expression in tissues and organs.

In recent years, with the increasing research on the molecular characterization of ALDH2 in the kidney, its role in disease has been emphasized. Increasing evidence suggests that ALDH2 is important in regulating oxidative stress, autophagy, and apoptosis in the kidney. Xu T et al. found that ALDH2 could regulate autophagic response through Beclin-1 signaling pathway to alleviate acute kidney injury (26). Kim J et al, on the other hand, found that the lack of ALDH2 activity in renal tissues aggravated renal tissue injury in cisplatin-induced renal injury by increasing the generation of ROS, enhancing cisplatin sensitivity and cytotoxicity (27). Chen et al. found that knockdown of ALDH2 in mouse kidney IRI would significantly aggravate the degree of renal function injury and increase the level of apoptosis, as well as exacerbate the inflammatory response of the kidney after renal IRI in mice (28). Thus, it is clear that the reduction of ALDH2 expression level is an important factor leading to the increased degree of renal injury, and increasing the expression of ALDH2 plays a crucial role in alleviating renal injury. We intervened with Alda-1, an ALDH2-specific agonist, to increase ALDH2 expression levels in renal tissues in the current study. We found that increasing the expression of ALDH2 significantly alleviated the degree of renal functional impairment after IRI in rat kidneys, the expression of the renal tubular injury marker NGAL and the apoptotic protein BAX was significantly reduced, and the expression levels of inflammatory factors (IL1β and IL6) were significantly reduced. This again demonstrates that ALDH2 has a protective effect against renal IRI.

With the above analysis, we demonstrated that ALDH2 has a protective effect against kidney injury. However, the potential mechanism of ALDH2’s role in kidney transplantation AKI is currently unknown to us. Therefore, we explored the level of change of 186 KEGG signaling pathways in AKI in the present study using ssGSEA enrichment analysis for ALDH2 and KEGG pathways using correlation analysis. The results indicate that ALDH2 correlates with the level of changes in multiple signaling pathways. Among them, the MAPK signaling pathway has attracted our attention.

The Mitogen-activated protein kinase (MAPK) signaling pathway is a series of highly conserved enzymatic response cascades that regulate many biological processes including: cell proliferation, differentiation, transformation, inflammation, and apoptosis (29). The current study found that MAPK is mainly categorized into four subfamilies, namely, ERK, P38, JNK, and ERK5 (30). Normally, MAPK is in a non-phosphorylated state and undergoes phosphorylation modification leading to enhancement of its activity when subjected to external stimuli. Inhibition of the MAPK signaling pathway reduces the expression of inflammatory factors and is one of the important mechanisms for alleviating kidney disease. Ma L et al. found that Baicalin could attenuate oxidative stress and inflammatory responses in diabetic nephropathy by inhibiting the MAPK signaling pathway (31). Chen L et al, on the other hand, demonstrated that trans-cinnamaldehyde could alleviate renal IRI by inhibiting the JNK/p38 MAPK signaling pathway and attenuating the inflammatory response (32). In addition to this, the relationship between ALDH2 and the MAPK signaling pathway has also attracted the attention of researchers. Zhong Z et al. found that ALDH2 was able to inhibit the MAPK signaling pathway to alleviate liver injury (33). Jin J et al, on the other hand, found that ALDH2 was able to inhibit the MAPK signaling pathway and ameliorate the level of oxidative stress and inflammation in LPS-induced AKI, thereby alleviating renal injury (34). However, so far, no study has reported the relationship between ALDH2 and MAPK signaling pathways in kidney transplantation AKI. Our present findings showed that ALDH2 was negatively correlated with the MAPK signaling pathway in kidney transplantation AKI, demonstrating that ALDH2 is also able to inhibit the activation of the MAPK signaling pathway in kidney transplantation AKI. We also verified the above conclusion using animal experiments that increasing the expression level of ALDH2 significantly inhibited the activation of the MAPK signaling pathway in rat kidney IRI. Therefore, we suggest that ALDH2 exerts its protective effects by inhibiting the MAPK signaling pathway.

In summary, we identified ALDH2 as a key gene for kidney transplantation AKI using WGCNA and machine learning algorithms. Combining bioinformatics and animal experiments, we found that ALDH2 may alleviate renal IRI by inhibiting the MAPK signaling pathway and reducing inflammatory responses. Our study provides a new therapeutic target for the treatment of AKI caused by ischemia-reperfusion during kidney transplantation. However, we recognize that this study has some limitations. First, in the bioinformatics analysis section, our analysis is a secondary mining of previously published datasets, which may lead to different conclusions due to different analytical ideas and perspectives; Second, in the animal experimental part, we utilized rat renal IRI to validate the potential role and mechanism of ALDH2, which is a model only for AKI caused by IRI during kidney transplantation and not a substitute for AKI caused by pharmacological factors or immune rejection; Finally, in the mechanism of action section, we only explored the potential role of the MAPK signaling pathway, and subsequent validation of other pathways is still needed.

## 5 Conclusion

The current study utilized WGCNA and machine learning algorithms (Random Forest, LASSO regression analysis and SVM algorithm) to identify ALDH2 as a key gene in kidney transplantation AKI. Combined with ssGSEA enrichment analysis and animal experiments, we found that ALDH2 may alleviate renal IRI by inhibiting the MAPK signaling pathway and reducing inflammatory responses. ALDH2 may serve as a novel target for mitigating kidney transplantation AKI, providing new options for the treatment of kidney transplantation AKI.

## Data Availability

The datasets presented in this study can be found in online repositories. The names of the repository/repositories and accession number(s) can be found in the article/**Supplementary Material**.
