# Supplementary material for: Identification of ALDH2 as a novel target for the treatment of acute kidney injury in kidney transplantation based on WGCNA and machine learning algorithms and exploration of its potential mechanism of action using animal experiments
Source: Front Immunol. 2025 Mar 4;16:1536800. doi: 10.3389/fimmu.2025.1536800 (PMC11913804; doi:10.3389/fimmu.2025.1536800)
Supplement: Supplementary file 1 [file DataSheet1.docx]

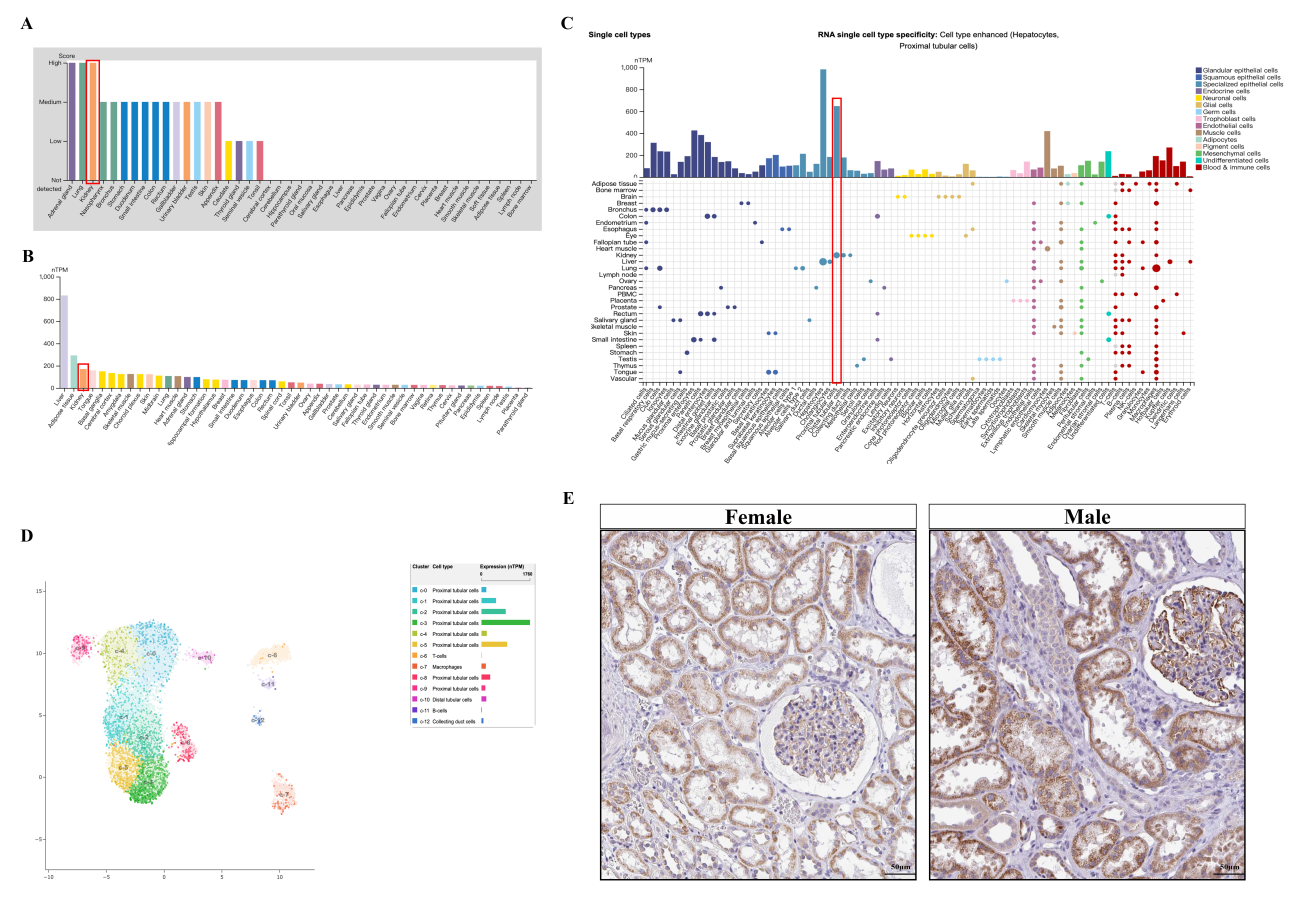


**Supplementary Figure 1. Expression landscape of Hub genes.** A represents the protein expression level of the Hub gene ALDH2 in each tissue and organ; B represents the mRNA expression level of the Hub gene ALDH2 in each tissue and organ; C represents the expression of the Hub gene ALDH2 at the cellular level; D represents the expression level of the Hub gene ALDH2 in single-cell sequencing of kidney tissue; E represents an immunohistochemical plot of ALDH2 expression levels in normal human kidney tissue.
